# How Can Selected Dietary Ingredients Influence the Development and Progression of Endometriosis?

**DOI:** 10.3390/nu16010154

**Published:** 2024-01-02

**Authors:** Monika Abramiuk, Paulina Mertowska, Karolina Frankowska, Paulina Świechowska-Starek, Małgorzata Satora, Grzegorz Polak, Izabela Dymanowska-Dyjak, Ewelina Grywalska

**Affiliations:** 1 Independent Laboratory of Minimally Invasive Gynaecology and Gynaecological Endocrinology, Medical University of Lublin, Staszica 16 St., 20-081 Lublin, Poland; grzegorz.polak@umlub.pl (G.P.); izabela.dyjak@gmail.com (I.D.-D.); 2 Department of Experimental Immunology, Medical University of Lublin, Chodźki 4a St., 20-093 Lublin, Poland; paulina.mertowska@umlub.pl (P.M.); ewelina.grywalska@umlub.pl (E.G.); 3 1st Chair and Department of Oncological Gynecology and Gynecology, Students’ Scientific Association, Medical University of Lublin, Staszica 16 St., 20-081 Lublin, Poland; k.frankowska10@gmail.com (K.F.); malgorzata.satora@gmail.com (M.S.); 4 1st Chair and Department of Oncological Gynaecology and Gynaecology, Medical University of Lublin, Staszica 16 St., 20-081 Lublin, Poland; pswiechowska.kontakt@gmail.com

**Keywords:** endometriosis, nutrition, vitamins, macronutrients, micronutrients, diet, phytoestrogens, xenoestrogens

## Abstract

Endometriosis is a chronic, hormone-dependent disease characterized by the presence of endometrial tissue in ectopic locations. Since the treatment options for this disease are still limited, and the cure rate is unsatisfactory, the search for ways to treat symptoms and modify the course of the disease is of key importance in improving the quality of life of patients with endometriosis. So far, the literature has shown that nutrition can influence endometriosis through hormonal modification and altering the inflammatory or oxidative response. Since the importance of nutrition in this disease is still a subject of scientific research, we aimed to summarize the current knowledge on the role of dietary modifications in endometriosis. Our review showed that nutrients with anti-inflammatory and antioxidant properties, including most vitamins and several trace elements, may influence the pathogenesis of endometriosis and can be considered as the nutrients preventing the development of endometriosis. However, despite the many discoveries described in this review, further interdisciplinary research on this topic seems to be extremely important, as in the future, it may result in the development of personalized therapies supporting the treatment of endometriosis.

## 1. Introduction

Endometriosis is a hormone-dependent chronic disease in which endometrial tissue develops outside the uterine cavity. It is widely accepted that the development and persistence of endometriosis depend on various immunological, hormonal, and genetic factors. Given this complexity, the condition requires a multifaceted approach that includes various supportive therapies [1].

Nutrition can influence a wide range of processes that underlie the causes of endometriosis. Since it has been proven that chronic inflammation and excessive oxidative stress occurring within ectopic lesions contribute to the development of the disease, nutrients influencing these processes are also factors modifying the course of the disease [1]. Moreover, recent discoveries have shown that the relationship between endometriosis and nutrition involves a kind of extensive reciprocal influence. A recent meta-analysis found an association between endometriosis and leptin, an adipokine released from adipose tissue that acts as an appetite regulator and mediates metabolic regulation. It is therefore possible that endometriosis can indirectly regulate food intake [2]. On the other hand, there is also a large amount of evidence supporting the importance of nutrition in modifying the symptoms of endometriosis. Certain foods, such as fruits, vegetables, and dairy products, may alleviate menstrual pain, which is a hallmark of the disease, by influencing inflammatory mediators [3]. Dietary interventions also play an important role in the treatment outcomes of patients suffering from infertility [4,5]. The current literature indicates the influence of overweight or underweight, the way food is processed, or the consumption of products with antioxidant and anti-inflammatory properties on various areas of infertility, including ovarian function and endometrial receptivity [5]. Although both the diverse pathogenesis of endometriosis and the multitude of dietary choices make it difficult to link all these relationships, it seems particularly important to determine which dietary restrictions or enrichments may be the most valuable, firstly because a significant percentage of patients with endometriosis decide to make various independent lifestyle modifications to alleviate the symptoms of the disease [6,7,8], and secondly due to the lack of clear recommendations included in the guidelines for the treatment of endometriosis [9].

Another relevant issue that needs to be addressed is the presence of various biologically active contaminants as the unintentional components of dietary products. The current literature suggests that substances like phthalates, dioxin, or bisphenol A (BPA) inadvertently contained in food may act as endocrine disruptors and in consequence can influence the course of the disease [10].

As new research emerges, there is an urgent need to develop new, timely conclusions that will shed new light on this extremely important issue. Therefore, this study discusses the impact of various dietary components, including the role of vitamins, micro and macro elements, as well as sources of estrogen derivatives found in the diet, on the course of endometriosis.

## 2. Materials and Methods

### Search Strategy, Study Selection, and Data Extraction

The literature analysis was performed in the PubMed and Web of Sciences databases, where the search for available articles was based on the keyword “endometriosis”. The available articles were then narrowed down based on the period from 2013 to 2023. The remaining articles were then filtered for the presence of keywords such as “diet”, “nutrition”, “vitamins”, and “minerals”. Articles with full access and available abstracts of paid publications were analyzed. The remaining articles were analyzed by the authors for inclusion in the publication. Among the found articles, duplicates were rejected at each stage of the analysis. The final number of included articles was 171. Of all the publications included in this manuscript, 81 were reviews and meta-analyses, 14 were studies on animal models, and 76 were research articles.

## 3. The Role of Selected Dietary Components in the Course of Endometriosis

**Figure 1:**
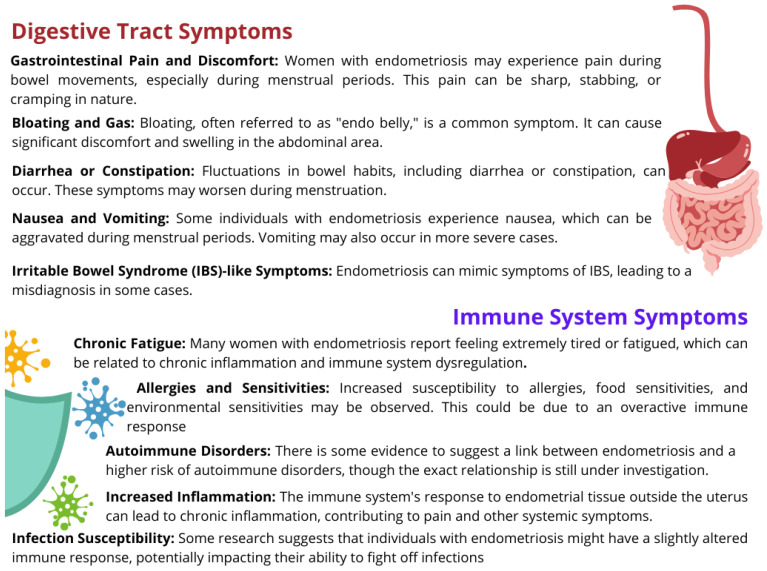
Examples of symptoms of the digestive tract and immune system observed in the course of endometriosis; based on [11,12,13].

Endometriosis can affect various systems in the body, including the digestive tract and immune system. Symptoms resulting from these systems can sometimes be complex and very diverse. From the gastrointestinal tract, these may include abdominal pain, diarrhea, constipation, nausea, or vomiting, which significantly limit the patient’s ability to function daily (Figure 1). However, on the part of the immune system, we experience the possibility of food allergies and intolerances, weakness or chronic fatigue, increased inflammation, and the risk of developing infections [11,12,13] (Figure 1). It is important to note that symptoms can vary greatly among people with endometriosis, and not everyone will experience all of these symptoms. However, most of these symptoms are largely related to the patient’s lifestyle, specifically their diet. The diet of patients contributes to the deepening of nutritional deficiencies in the body [14].

Nutrient deficiencies may have a significant impact not only on the occurrence of endometriosis but also by exacerbating symptoms or affecting the progression of the disease [14]. The most frequently observed nutritional deficiencies in patients with endometriosis concern magnesium, iron, B vitamins (especially B6 and B12), zinc, selenium, and folic acid [15,16]. The impact of nutrient deficiencies on endometriosis can be multifaceted, affecting pain, inflammation, immune function, and overall quality of life. Therefore, a diet rich in essential nutrients, possibly supplemented with vitamins and minerals when necessary, can play a key role in the treatment of endometriosis [16,17]. However, supplementation should be directed by a health care professional, as excessive consumption of certain nutrients can also have adverse effects. Regular monitoring and individualized nutritional advice from a healthcare professional or registered dietitian can help effectively treat these aspects of endometriosis. Therefore, a properly adapted and balanced diet in the treatment of endometriosis should focus on eliminating nutritional deficiencies, eliminating excess estrogen, reducing inflammation, reducing oxidative stress, and reducing exposure to estrogen derivatives (phytoestrogens and xenoestrogens) [18,19,20].

### 3.1. The Importance of Vitamins in Endometriosis

#### 3.1.1. Vitamins C and E

In the current literature, much attention is paid to the antioxidant properties of vitamins C and E. As their synergic effect of action is reported, the possible benefits from their combined use can be expected [21]. Vitamin C, also called ascorbic acid, has documented antioxidant properties [22], serves as a cofactor for many essential enzymes, is involved in the synthesis of catecholamines and vasopressin [22], and is involved in the process of collagen hydroxylation [23,24]. Vitamin E is known primarily as an antioxidant with additional antiangiogenic and anti-inflammatory effects [25,26]. Because both vitamins are involved in antioxidant processes, which are also the basis of the pathogenesis of endometriosis, many researchers, apart from assessing the separate effects of these vitamins, have focused on their combined effect.

In the case of vitamin E, significant discrepancies can be observed, which are difficult to clearly explain. First, the study by Da Broi et al. found higher concentrations of vitamin E in the follicular fluid of patients with endometriosis compared to healthy women [27]. An increased share of vitamin E in the mechanism of activation of its antioxidant properties was suggested as a potential explanation for this condition. On the other hand, many authors either did not notice a relationship between the level of vitamin E and the occurrence of the disease [28,29,30] or even observed a reduced concentration of this microelement in serum [31]. Referring to the causes of reduced vitamin E levels, we propose a hypothesis suggesting that this condition is the result of consuming a certain amount of vitamin E in antioxidant processes. Results regarding the assessment of vitamin C levels are also inconsistent. Lu et al. found that a lower vitamin C content in follicular fluid is characteristic of endometriosis [32]. However, Nishihara et al. did not observe a relationship between the concentration of vitamin C in follicular fluid and the occurrence of endometriosis [33].

However, increasing evidence from animal studies indicates the inhibitory effect of vitamin C supplementation on lesions associated with endometriosis [34,35,36,37]. In such experiments, investigators used various parameters to describe the appearance of ectopic endometrial tissue, such as the size, volume, and mass of the lesions, as well as various assessments related to associated adhesions, assessment of fibrosis, and the condition of the mucosa associated with the lesion epithelium. Although all authors consistently observed the inhibitory effect of vitamin C, which was reflected in a decrease in the above-mentioned parameters describing the lesions, the only parameter in all the analyzed studies that underwent significant changes in the groups supplemented with vitamin C was the volume of lesions [34,35,36,37].

Moreover, the effect of the combined administration of vitamins C and E was also assessed in human models. Such supplementation resulted in pain relief [38,39] and was able to reduce both the level of oxidative stress and inflammatory markers [38,39]. The fact that vitamin C supplementation alone did not affect molecules indicative of oxidative stress, including reactive oxygen species (ROS), superoxide dismutase (SOD), and malondialdehyde (MDA), may support the more beneficial use of vitamins C and E together [32].

The intake of vitamins C and E was also assessed for the risk of endometriosis [15,40,41]. In this case, although vitamin C has been mainly presented as a micronutrient providing a protective effect against endometriosis [15,40,41], most studies have shown that vitamin E is not associated with the risk of the disease [15,41].

#### 3.1.2. Vitamin D

Vitamin D acts mainly as a modulator of metabolic reactions and immune response [42]. Since the endometrium is a tissue susceptible to changes in secretory activity under the influence of vitamin D [43], the question remains whether there is a relationship between vitamin D and endometriosis.

**Figure 2:**
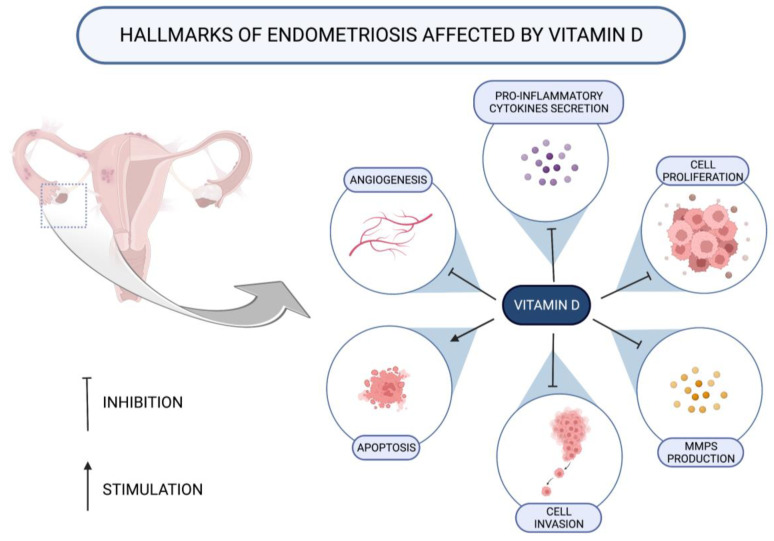
Different mechanisms of action of vitamin D on endometriotic lesions; based on [44,45,46,47,48,49,50,51,52].

The treatment of ectopic endometrial cells with vitamin D within in vitro studies, as well as the use of induced endometriosis in animal models, revealed several possible substantial pathways for affecting the disease (Table 1; Figure 2).

Several of the conducted animal studies found vitamin D to reduce the size or volume of endometriotic lesions [44,45,46].

Furthermore, it has been noticed that vitamin D has the potential to interact with proinflammatory cytokines and other molecules intermediating in inflammatory processes. Thus, under the influence of vitamin D, the production of interleukin-17 (IL-17) [44] and IL-6 decreases [47,50]. Additionally, the experimental study conducted by Miyashita et al. found that ectopic endometrial stromal cells (EESCs) incubated with 1,25-(OH)_2_D_3_ were characterized with significantly lower amounts of IL-8, cyclooxygenase-2 (COX-2) mRNA, and prostaglandin E2 (PGE2) [48].

Vitamin D can also act as a disruptor of matrix metalloproteinase (MMP) activity. The concentrations of both MMP-2 and MMP-9 were reported to be lower under the influence of 1,25-(OH)_2_D_3_ [46,48], probably partially as the result of boosting the pathways involved in MMP inhibition [46,49]. Such action may contribute endometriosis reduction through decreasing of invasion and proliferation of endometriosis lesions [53].

On the other hand, this biomolecule is also able to alleviate angiogenesis, as both the reduced expression of vascular endothelial growth factor-A (VEGF-A) genes [50] as well as inhibition of the nuclear factor kappa B (NF-κB) pathway participating in neovascularization [48] were found in two studies on cellular models.

Decreasing invasiveness of endometrial cells in an environment rich in vitamin D has been observed [50,51]. Further, the decreased cell proliferation activity was explained by the contribution of vitamin D in reducing the activity of several pathways essential for the occurrence of the process [48,49,50,54]. Hence, much attention was paid to the disruption of the Wnt/β-catenin pathway functioning by reducing catenin activity [55]. This pathway was also affected by the following changes regarding NF-κB, the protein complex involved in its course [55]: in the endometriotic cells incubated with 1,25-(OH)_2_D_3_, IκBα, an inhibitor of NF-κB was preserved, thus extinguishing the NF-κB-related proliferation [48]. Contrastingly, the data regarding apoptosis are inconclusive; while some researchers did not observe pro-apoptotic values of vitamin D [48,50], Abbas et al. [45] and Rashidi et al. [52] suggested inhibition of apoptosis as a significant action of vitamin D. Most of the studies evaluating apoptosis were designed as in vitro studies and used similar molar concentrations of vitamin D [48,50,52]; therefore, it is difficult to show where the differences in the results came from and to translate these doses into doses suitable for supplementation in humans.

Many studies conducted on patients with endometriosis also focused on measuring the concentration of vitamin D in plasma and peritoneal fluid. These reports were largely consistent, as most researchers observed reduced levels of vitamin D in plasma [48,56,57,58] and peritoneal fluid [56,57] in women with this disease, and only a few authors did not observe statistically significant differences [51,59,60]. Additionally, it has been proposed that vitamin D deficiency can lead to larger ovarian endometriosis lesions [61].

Another widely discussed issue was the impact of vitamin D supplementation on the pain associated with endometriosis. Since it has been shown that in women with endometriosis, the occurrence of severe pain correlates with vitamin D deficiency, the role of this vitamin in pain relief can be expected [58]. In a group of patients with endometriosis [56,62], vitamin D supplementation alleviated the pain. Nevertheless, some authors found no correlation between vitamin D supplementation and the reduction of dysmenorrhea [63,64]. In studies in which the analgesic effect of vitamin D was not observed, adolescent patients were also included in the study groups [63,64]. Therefore, we suspect that the reason for such observations may have been the different and more severe nature of pain often observed in adolescents [65].

In conclusion, numerous in vitro and animal studies comprehensively demonstrate the mechanisms of vitamin D action, suggesting a significant role for this vitamin in the development of endometriosis. However, further studies in humans are needed to confirm these reports.

#### 3.1.3. Vitamin A

The results of recent studies aimed at comparing the levels of various forms of vitamin A in normal and endometriotic tissues are not numerous, and they mainly focus on the role of all-trans-retinoic acids (ATRA) in in vitro models.

Pierzchalski et al., in their study, directly assessed the level of retinoids in ectopic and eutopic tissues of women with endometriosis. While they observed higher concentrations of retinol and retinyl esters in endometrial lesions, ATRA levels tended to be lower [66].

The importance of ATRA in the pathogenesis of endometriosis was also discussed in terms of ATRA’s ability to contribute to hormonal changes and the resulting inhibitory effects on endometrial tissue. In an in vitro study, the effects of incubation of isolated endometriosis stromal cells with ATRA were assessed. The authors, in their experiment, used ATRA in 10^−7^ M concentrations. In the course of the study, two important observations were in the course of the therapy [67]. Firstly, an increasing mRNA expression of various genes associated with an inhibitory effect on cell proliferation was observed, and secondly, a simultaneous increase was seen in the expression of HSD17B2 mRNA, responsible for the conversion of estradiol to its less biologically active form—estrone [67,68]. Although the reduction in estradiol concentrations after exposure to ATRA was not statistically significant, because this form of vitamin A has been shown to affect important targets in the pathogenesis of endometriosis, we consider its role in the disease to be promising [67].

In addition to reducing hormonal secretion, the inhibitory effect of ATRA exerted on endometrial lesions also appears to involve the suppression of IL-6, which is involved in the pathogenesis of endometriosis [69]. This interaction of retinoic acid with IL-6 was demonstrated in another in vitro study. It was observed that ATRA at a concentration of 10^−6^ M reduced the level of IL-6 and, through reducing this interleukin, inhibited processes linked to epithelial-to-mesenchymal transition (EMT), including migration and invasion of endometriotic cells [70].

Although the results regarding incubation of endometrial cells with ATRA are very promising [67,70], the concentrations of ATRA used in the above-mentioned studies were much higher than concentrations of ATRA detected in human liquids and tissues [71]. Therefore, it is important to approach the above-mentioned results with caution.

#### 3.1.4. B Vitamins

The B vitamin group consists of eight water-soluble vitamins, including thiamine (vitamin B1), riboflavin (vitamin B2), niacin (vitamin B3), pantothenic acid (vitamin B5), pyridoxine (vitamin B6), biotin (vitamin B7), folic acid (vitamin B9), and cobalamin (vitamin B12). Because these vitamins form such an extensive family of compounds, they are found in almost all food products [72]. It is difficult to standardize the function of B vitamins, but it is known that all of them are involved in various cellular processes, including both catabolic and anabolic ones [72,73]. Analyzing research focusing on the role of B vitamins in the context of reproductive health, most reports focus on the impact of these vitamins on the proper development of the fetus [74]. Although there are no studies on the influence of B vitamins on the formation and development of endometriosis, existing studies have attempted to find such relationships.

There are two studies examining the relationship between B vitamin intake and the incidence of endometriosis. While one of them noted the protective effect of vitamins B1 and B9 from food against the development of the disease [40], the other pointed out such an effect of vitamins B2, B6, B9, and B12 [15].

In the context of endometriosis, it is worth mentioning that B vitamins have also been discussed as a possible antidote to period-related symptoms. Interestingly, vitamin B1, apart from helping to alleviate dysmenorrhea [75], is also described as a supplement that has a significant impact on eliminating the symptoms of premenstrual tension, both mentally and physically [76].

Although the presented results in terms of pain symptoms are largely encouraging, none of these studies were conducted on patients with endometriosis; therefore, further research on this group of patients is necessary.

### 3.2. The Importance of Macroelements in the Course of Endometriosis

**Figure 3:**
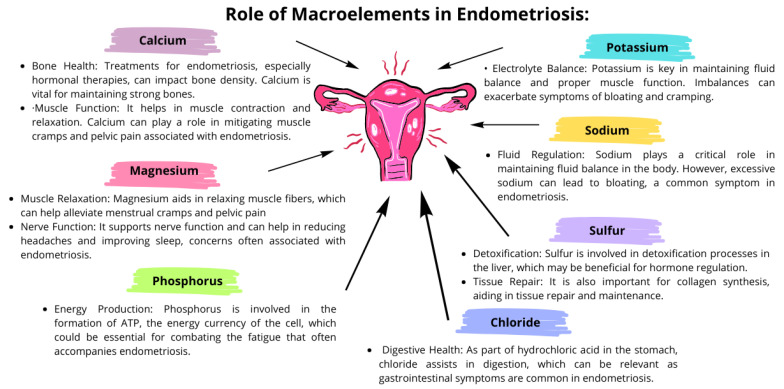
The importance of selected macroelements in the course of endometriosis; based on [77,78,79,80].

Macroelements, also known as macrominerals, play significant roles in the overall health and well-being of individuals, and they can have specific impacts on the course of endometriosis [77,78,79,80]. These elements, required in larger amounts by the body, include calcium, magnesium, sodium, potassium, chloride, phosphorus, and sulfur. Detailed interactions of these macroelements in the course of endometriosis are presented in Figure 3.

A balance of these macronutrients is essential not only for overall health but also for managing the symptoms of endometriosis. Deficiencies or imbalances can exacerbate symptoms such as pain, inflammation, bloating, and fatigue. To maintain the appropriate level of these macroelements, a properly balanced diet is important, possibly supplemented under the supervision of a doctor. It is also important to remember that the relationship between macronutrients and endometriosis may be complex. Diet, absorption problems, and the impact of endometriosis treatment can affect the levels of these macronutrients in the body. Therefore, the treatment of endometriosis often requires a holistic approach that includes nutritional support, treatment, and lifestyle modifications.

### 3.3. The Importance of Microelements in the Course of Endometriosis

Microelements, also known as trace minerals, are essential nutrients needed in smaller amounts than macroelements but are crucial for various bodily functions [78,81]. Their role in the course of endometriosis is significant due to their involvement in hormonal balance, immune function, inflammatory processes, and overall cellular health. The most important micronutrients influencing the development and progression of endometriosis are zinc, copper, iron, selenium, manganese, iron, and chromium [15,82,83].

#### 3.3.1. The Importance of Zinc

In general, zinc is responsible for maintaining the homeostasis of the organism through being a pillar of the proteins involved in various building, enzymatic, and catalysis processes; it is also presented under the form of ions acting as a signaling molecule [84].

The multifactorial background of endometriosis gives space for discovering possible links between zinc and the occurrence of the condition [85].

Increased migration, enhanced invasiveness, and resistance to apoptosis of endometrial cells in endometriosis are a result of epithelial-to-mesenchymal transition (EMT)—a process in which cells gradually lose their epithelial features and gain mesenchymal ones [86]. The regulation of EMT is mediated by several transcription factors (e.g., Snail, Slug, zinc-finger E-box-binding homeobox 1 (ZEB1), ZEB2, or Twist) [86,87], among which ZEB1 and ZEB2 contain a zinc molecule in their structure [88]. Additionally, during the EMT process, the extracellular matrix (ECM) degradation takes place through the action of a wide range of proteolytic enzymes representing the MMP family [86], also containing zinc as their component [89]. Thus, this micronutrient seems to be, in a multifaceted manner, involved in forming the endometriosis environment.

The majority of studies published so far consistently support the above-mentioned dependencies by indicating the greater expression of the following MMPs or their mRNA as measured in the eutopic and ectopic endometrium, serum, or follicular fluid of patients with endometriosis compared to healthy women: MMP-2 [90,91] and MMP-9 [91,92]; moreover, the positive relationship between MMP concentrations and the severity of endometriosis was also suggested [93]. MMPs are known to be susceptible to hormonal regulation, with increased action observed in greater estrogen concentrations [90] and inhibition under conditions of progesterone activity [91]. Thus, despite the reports that zinc deficiency may affect MMPs [94,95], it seems that in endometriosis, the altered hormonal balance may have a pivotal role in the regulation of MMP management when compared to zinc balance.

On the other hand, the association of other Zn-requiring molecules, ZEB1 and ZEB2, which are indisputably involved in the EMT process [86,87], with endometriosis is much less well investigated. While some researchers have noticed a positive association between ZEB1 and ectopic endometrial lesions [96], others have not observed any relationships [97]. Additionally, we found no reports suggesting that Zn deficiency may impair ZEB1 and ZEB2 expression.

The possible mechanisms of action of zinc described above constitute a key element in understanding the role of this trace element in endometriosis. In addition, there are studies directly examining the role of Zn in patients with endometriosis, which also constitute an important source of knowledge. In the studies by Messala et al. and Lai et al., the authors compared blood zinc level measurements in women with endometriosis and healthy control women [98,99]. Both authors found lower serum zinc concentrations in patients with endometriosis compared to the control group, by 22% [98] and 43% [99], respectively. Zinc levels were also lower in the follicular fluid of patients with endometriosis compared to those with tubal infertility. Another interesting observation of this study was the increased level of zinc in follicles in women with endometriosis who had a successful IVF pregnancy compared to patients who did not become pregnant [91]. Such a correlation may be indirectly related to the previously established important role of zinc in oocyte maturation [100] and fertilization [101].

Overall, although the role of zinc has been evaluated in a multifaceted manner and the results of the above-mentioned studies are consistent, there are still not enough studies to conclusively clarify it.

#### 3.3.2. The Importance of Copper in Endometriosis

Due to the presence of Cu in most food products, a standard, well-balanced diet covers the demand for this mineral. The food groups especially rich in this trace metal are meat products, including offal, as well as nuts and seeds. Although it is known that excess Cu is highly toxic, this is rare, because even in the above-mentioned foods, the amounts of this element are traces, and the system involved in the transport and storage of copper functions efficiently [102].

Looking at the body’s homeostasis as a whole, Cu is involved in several reactions as a component of the following enzymes: SOD, COX, diamine oxidase (DAO), and skin lysis oxidase (SLO) [102]. Importantly, reports suggest that the Cu/Zn-SOD type plays a major role in antioxidant protection in patients with endometriosis [103]. Additionally, by acting as a metalloestrogen, Cu can influence estrogen-dependent conditions [104]. In a study by Thézénas et al., the aim was to measure the concentration of copper-containing amine oxidase-3 (AOC3), an enzyme responsible for inducing ROS production and interfering with immune responses, in ectopic and eutopic endometrium. They found that elevated AOC3 levels correspond to an ectopic origin of the endometrium [105].

Furthermore, the current literature suggests positive correlations between Cu and markers of oxidative balance, including total antioxidant status (TOS) and oxidative stress index (OSI) [106]. Although the relationship between the mentioned enzymes involved in maintaining the oxidative balance and Cu, in general, seems unquestionable, the relationship of these enzymes with dietary copper requires further investigation [107].

The assessment of the role of Cu in endometriosis is multifaceted, ranging from dietary Cu intake to the content in various tissues or excretions, and is often enriched by the analysis of Cu-dependent oxidative stress markers. However, based on the available research results in the literature, the comparison of endometriosis patients with a healthy control group resulted in relatively inconsistent observations, in which the following were observed: increased Cu concentration in urine [108] and blood [106] in patients with endometriosis, and the lack of significant correlations of Cu measured in blood [99] and follicular fluid [91] with the occurrence of the disease. There are also reports discussing the possibility of Cu’s involvement in the therapeutic regimen of endometriosis. First, the combined use of Cu and curcumin was investigated. Although the addition of Cu effectively enhanced the effects of curcumin, in this case, this trace metal should be considered as an effective carrier rather than an active substance [109]. On the other hand, it was found that pharmacological reduction of copper levels may result in stopping the growth of endometriotic lesions. However, since the study was conducted in an animal model, these results cannot be interpreted for humans [82].

#### 3.3.3. The Role of Iron in Endometriosis

At the cellular level, Fe serves as a key molecule in the process of DNA synthesis and respiratory chain reactions; however, in the process of electron exchange, Fe may also contribute to the formation of ROS as a result of the transformation of Fenton reaction products [110]. Fe is therefore responsible for the phenomenon of oxidative stress—one of the pillars of endometriosis [111]. Since this disease is characterized by high iron content in the local environment due to retrograde menstruation, there is a high probability of oxidative stress [112]. Large amounts of this trace element have been detected not only in ectopic lesions [105,113,114] but also in peritoneal fluid [115], follicular fluid [91,116,117], and blood [118] collected from patients with endometriosis. Additionally, suggestions indicating the involvement of Fe in the malignant transformation of OMA [119], dysmenorrhea [120], or the formation of adhesions [121] further emphasize the important role of Fe in the pathogenesis of endometriosis. Unfortunately, despite such an important role of Fe in the course of endometriosis and numerous studies assessing its impact on the development of the disease, no reports link Fe with diet. Dietary assessment of Fe intake using the FFQ did not show significant differences in the intake of this micronutrient between women with endometriosis and healthy women [41]. Therefore, further research on this issue is needed to demonstrate whether exogenous dietary Fe can modify Fe metabolism and influence endometriosis.

#### 3.3.4. The Importance of Selenium in Endometriosis

Selenium (Se) is widely present in almost all food products, where it occurs in four main chemical forms: selenomethionine and selenocysteine, found in animal products, or selenate and selenite, found in foods of plant origin [122,123]. In the human body, selenium is an essential component of selenoproteins, which function as both enzymes and non-enzymatic proteins [124,125]. In the context of endometriosis, one such enzymatic protein in particular, glutathione peroxidase, seems important due to its involvement in the regulation of oxidative stress [124].

The only observed association of Se with the occurrence of the disease concerns the assessed properties of follicular fluid. First, Singh et al. observed that lower follicular fluid Se concentrations were associated with an increased risk of endometriosis-related infertility compared to tubal infertility. Additionally, since they found that selenium is positively associated with glutathione peroxidase, it may suggest a possible mechanism of the involvement of this trace metal in the pathogenesis of diseases, i.e., by impairing the sphere of oxidative stress [91].

#### 3.3.5. The Importance of Manganese in Endometriosis

Manganese (Mn) is an essential component of the following several enzymes: arginase, Mn-SOD, glutamine synthetase, and pyruvate carboxylase, which perform diverse essential functions in human organisms. With regard to the functioning of Mn-SOD, including its involvement in neutralizing free radicals as well as the pathway by which this enzyme is activated, which is under the influence of elevated levels of tumor-necrosis factor-alpha (TNF-α), the factor contributing to the maintenance of the inflammatory state in endometriosis, the association between Mn and endometriosis occurs [126].

The results regarding Mn-SOD activity in patients with endometriosis showed lower enzyme activity among patients with endometriosis when compared with the normal endometrium of healthy controls [103,127].

On the other hand, the assessment of Mn concentrations in blood [99] and urine [108] obtained from women with endometriosis did not bring any substantial reports. Similarly, the manganese intake did not differ significantly between patients with the disease and healthy controls [41]. Therefore, it seems that despite the presence of Mn in the Mn-dependent SOD, this trace metal alone does not play a major role in the disease.

#### 3.3.6. The Importance of Nickel in Endometriosis

Such a wide distribution of Ni in the environment (cereal products, green vegetables, nuts, and everyday metal products) has become problematic in light of frequent allergic reactions to Ni, causing various symptoms in various organs [128,129,130]. Ni allergy and endometriosis were assessed in two studies conducted by the same research group on large cohorts of patients with endometriosis: 7259 and 997 subjects, respectively. The frequency of Ni allergy was twice as high among women with endometriosis compared to women without this disease [131,132]. Although these observations suggest altered sensitivity to Ni among patients with endometriosis, observations regarding Ni concentrations in blood and urine in women with endometriosis are inconsistent. Although higher levels of Ni in the blood were detected in women with endometriosis compared to the control group [133], Pollack et al., in their ENDO study, did not observe any relationship between Ni concentration measured in urine and the risk of the disease [108].

Interesting results were obtained when examining the effect of a low-Ni diet on symptoms in women with both endometriosis and Ni allergy. In an open-label pilot study, a group of 31 patients with endometriosis and comorbid allergic contact mucositis (ACM) caused by Ni hypersensitivity were supplemented with a low-Ni diet for three months. It was examined whether such dietary modification could have an impact on the symptoms experienced by the participants. Two groups of symptoms were studied: gastrointestinal, including abdominal pain, nausea, and bobororhygma, as well as gynecological symptoms, including chronic pelvic pain, dysmenorrhea, and dyspareunia. Although the final results are promising, no conclusions can be drawn due to the small sample size and doubts as to which of these symptoms are characteristic of endometriosis and which are a manifestation of Ni hypersensitivity [134].

In conclusion, since the studies described above were designed to assess the relationship between nickel hypersensitivity or nickel-related allergy, rather than the role of nickel per se, no firm conclusions can be drawn regarding the role of nickel in endometriosis.

#### 3.3.7. The Importance of Chromium in the Course of Endometriosis

Whether chromium (Cr) constitutes an essential micronutrient or rather should be treated as a trace metal able to modify glucose metabolism has not been elucidated so far [135]. The distribution of Cr in various dietary products is quite similar, but still, there are no reports describing chromium deficiency in humans [136,137].

Possibly due to the above-mentioned Cr characteristic, the analysis of its role in endometriosis is superficial, and no substantial reports considering this topic in connection with diet are available. While Lai et al. did not observe differences in chromium assessed in serum between patients suffering from endometriosis and healthy controls [99], the results of the ENDO study led by Pollack et al. indicate higher Cr levels as a risk factor of endometriosis occurrence [108]. Thus, taking into consideration the small number of studies, no substantial conclusions regarding this micronutrient can be drawn.

### 3.4. The Importance of Fatty Acids in Endometriosis

Food flakes constitute a heterogeneous group of nutrients, and their classification includes several different divisions. Firstly, depending on their origin, animal and vegetable fats can be distinguished. Moreover, taking into account the more detailed structure of the molecules, they include omega-3 polyunsaturated fatty acids (omega-3-PUFAs), omega-6 polyunsaturated fatty acids (omega-6-PUFAs), saturated fatty acids (saturated-FAs) and trans fatty acids (TFAs) [138]. The main representatives of omega-3-PUFA are α-linolenic acid (ALA), eicosapentaenoic acid (EPA), and docosahexaenoic acid (DHA), and the two main components of omega-6-PUFA are linoleic acid (LA) and arachidonic acid (AA) [139]. Omega-3-PUFA and omega-6-PUFA, in particular, due to their ability to modulate inflammatory processes, are expected to play a significant role in regulating the development of endometriosis [140].

#### Omega-3-PUFA

To date, the largest number of studies have examined the effect of omega-3-PUFA supplementation on endometriosis, likely given their known anti-inflammatory potential [139] (Table 2).

The assessment of the impact of exclusively omega-3-PUFA supplementation in animal endometriosis models supported the multifarious effects of such a diet regimen. The inclusion of this group of dietary fats into supplementation was a trigger for the reduction of pro-inflammatory cytokines such as IL-6 and TNF-α and the level of VEGF in peritoneal fluid [47].

The impact of omega-3-PUFAs on endometriotic lesion size and inflammatory mediator production was also investigated in studies, in which transgenic mice models able to convert omega-6-PUFAs to omega-3-PUFAs were used [141,142]. In these organisms, several abilities of omega-3-PUFA action on endometriotic foci have been presented, including the reduction of endometrial lesions, lowering of the levels of IL-6 [141], and the reduction of COX-2 and Ph-3-mitotic marker expression. Such decreased expression of molecules involved in tissue proliferation suggests an inhibitory effect of omega-3-PUFA supplementation on endometriosis development [142]. Furthermore, since the current literature suggests the high utility of transgenic mouse models in evaluating the role of omega-3-PUFAs in humans, we believe there is potential to transfer these results to human models [144].

Another interesting beneficial effect of omega-3-PUFA supplementation concerns the impact of such a diet modification on suppressing endometriosis-related adhesion development. This action of omega-3-PUFAs has been explained by the influence of these fatty acids on collagen distribution [143]. Such an observed relationship should be deemed especially important due to the data indicating adhesions as a substantial factor that can deteriorate the quality of patients’ lives [145].

The results of studies performed to assess the various omega-3-PUFA and omega-6-PUFA concentrations in tissues obtained from humans [146,147] are also worth mentioning. However, the significant results refer only to EPA, and its lower serum concentrations were found in patients with endometriosis [146].

One of the more disappointing observations found during our literature review was the lack of effect of omega-3 supplementation (EPA and DHA) on pain symptoms of patients with endometriosis. In this study, the omega-3-PUFAs were contained in fish oil, and the concentrations of EPA and DHA in this product were known before supplementation [63]. However, since this is only one study focusing on endometriosis symptoms, it is not possible to juxtapose these results and verify their reliability.

### 3.5. The Importance of Carbohydrates

Due to the wide distribution of various carbohydrates in food, studies assessing their importance in endometriosis are methodologically difficult. This common occurrence of carbohydrates was also reflected in the results of population studies, as observations regarding total carbohydrate intake and endometriosis did not indicate any relationship [41,148,149].

However, the relationship between individual groups of carbohydrates and the disease has already been observed, as confirmed by Schink et al. in their retrospective case-control study, which found lower average maltose and glycogen intake in patients with endometriosis compared to healthy women [41].

Generally, the most frequently analyzed carbohydrate fraction was fiber; however, the obtained results are not conclusive. Youseflu et al. reported that total fiber intake did not correlate with the risk of endometriosis [148]. Similarly, Schwartz et al., in a prospective study conducted within the NHS II cohort, also found no association between total fiber and the risk of endometriosis. On the other hand, they found a higher risk of endometriosis associated with the consumption of total plant fiber and cruciferous plants, as well as a reduced risk of disease as a consequence of the consumption of fruit fiber. Another interesting observation they made related to carbohydrates was the higher risk of endometriosis from eating foods with a high glycemic index. However, no other data are available to compare with these conclusions [149].

### 3.6. The Importance of Protein in Endometriosis

Dietary protein can reduce the level of inflammatory markers in some inflammatory diseases [150,151,152]. This mechanism of action, as well as the effect on reducing retrograde menstruation due to the high magnesium content, seems to be responsible for the positive role of a diet rich in dairy products in endometriosis [16].

Much of the evidence on the effect of protein intake on endometriosis was summarized in a recently published meta-analysis [153]. In addition to finding a correlation between total dairy product intake and the risk of endometriosis, another goal of the authors was to assess the dose–response relationship between different groups of dairy products and the risk of the disease. For total dairy, high-fat dairy, and cheese intake, they noted a reduced risk of endometriosis due to increased consumption of these food groups when intake exceeded 21 (95% CI 0.76–1.00), 18 (95% CI 0, 76–0.96), and 2 (95% CI 0.79–1.00) servings per week. Such a dose-dependent correlation was also observed in the case of total milk consumption—18 servings (95% CI 0.80–0.9-) per week—but unlike the above fragments, the qualitative analysis did not reveal any correlations. However, for other dairy product groups, including low-fat dairy, whole milk, ice cream, low-fat milk, and yogurt, no correlations were observed. Based on these results, it can be concluded that dairy products with a higher fat content had a more beneficial effect [153]. We hypothesize that the potential explanation for this association may be the phenomenon of estrogen dissolution in adipose tissue [154].

A case-control study, not included in the meta-analysis, conducted by Schink et al. addressed a similar topic. Nevertheless, it is difficult to compare their observations with the statements from the meta-analysis, because they proposed testing protein fractions that had not been previously taken into account. They found a lower intake of animal protein in patients with endometriosis compared to the control group, suggesting a potential relationship between this group of dairy products and the development of the disease. Conversely, no relationship was observed between the plant fraction and total consumption of dairy products [41]. However, since plant products can be a source of pesticides [155], the lack of beneficial effect of plant protein on the course of endometriosis does not seem surprising. It is known that pesticides, such as organophosphorus or pyrethroid, can act as endocrine disruptors and increase endometriotic lesions [156]. Therefore, it could be even expected that increased consumption of vegetable protein would exacerbate the disease.

The study by Youseflu et al. sought to assess differences in sleep quality and lifestyle factors, including diet, between women with endometriosis and healthy women. Their observations regarding the relationship between diet and endometriosis are consistent with the conclusions of the meta-analysis [153], as they noticed lower consumption of dairy products in women with endometriosis compared to the control group [157]. However, it should be noted that this study was conducted with the same study group as one of the studies included in the meta-analysis, so equivalent results were expected [148].

Other interesting results on the role of protein intake in women suffering from endometriosis were reported in the study by Yamamoto et al. Although the principal objective of this prospective cohort study was to evaluate the impact of meat consumption on endometriosis development, the authors also noticed several essential findings regarding animal-derived proteins. Firstly, among products rich in proteins, including poultry, fish, shellfish, or eggs, only increased consumption of the first product was associated with higher risk of endometriosis. On the other hand, replacing red meat with fish, shellfish, or eggs was found to reduce the risk of the disease. Hence, it can be concluded that consumption of protein-rich products other than meat has a beneficial impact on endometriosis prevention [158].

### 3.7. The Influence of Estrogen Derivatives on the Development and Progression of Endometriosis

Estrogen plays a key role in the development and progression of endometriosis; therefore, estrogen derivatives may have a significant impact on this disease. Understanding the effects of these derivatives is crucial for the effective treatment of endometriosis.

#### 3.7.1. Effect of Phytoestrogens

**Figure 4:**
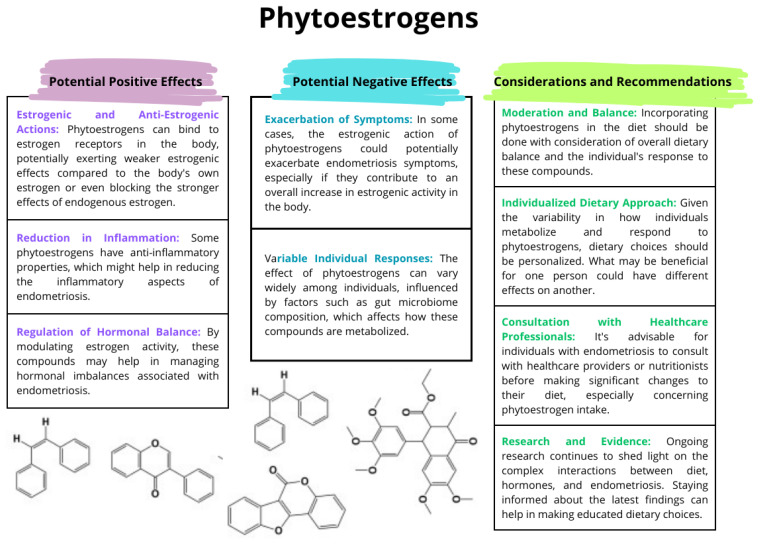
The effect of phytoestrogens on endometriosis; based on [159,160,161,165,166].

Dietary estrogen derivatives, often referred to as phytoestrogens, are plant-derived compounds that can mimic or modulate the effects of estrogen in the human body. Their impact on endometriosis is the subject of constant research and discussion because, depending on various factors, they may have both a positive and negative impact on its condition (Figure 4) [159,160,161]. Currently, there are three types of phytoestrogens: isoflavones, lignans, and coumestans. The first is found mainly in soy products such as soybeans, tofu, tempeh, and soy milk. Isoflavones are the best-studied group of phytoestrogens. Examples are genistein and daidzein. The second group of phytoestrogens is present in large amounts in linseed and sesame seeds and in smaller amounts in cereals, vegetables, fruits, and some drinks. Examples include secoisolariciresinol and matairesinol. The third group is less common and occurs in some legumes and sprouts. An example is coumestrol [162,163,164]. Phytoestrogens can bind to estrogen receptors in the body. There are two main types of estrogen receptors, ERα and ERβ, and phytoestrogens have a preference for ERβ. The binding can either mimic estrogen (estrogenic effect) or block estrogen from binding (anti-estrogenic effect). They may also influence the body’s production of natural estrogen, either by modulating the activity of enzymes involved in estrogen metabolism or by affecting the overall hormonal balance [159,165].

Phytoestrogens continue to be the subject of scientific research due to their potential health benefits and implications. Their effects may vary depending on the type of phytoestrogen, its amount, individual metabolism, and overall diet. Phytoestrogens can potentially influence the course of endometriosis; their effects are not simple and may vary from person to person. Understanding and monitoring individual responses to these dietary components is crucial in determining their role in the treatment of endometriosis.

#### 3.7.2. Effect of Xenoestrogens

**Figure 5:**
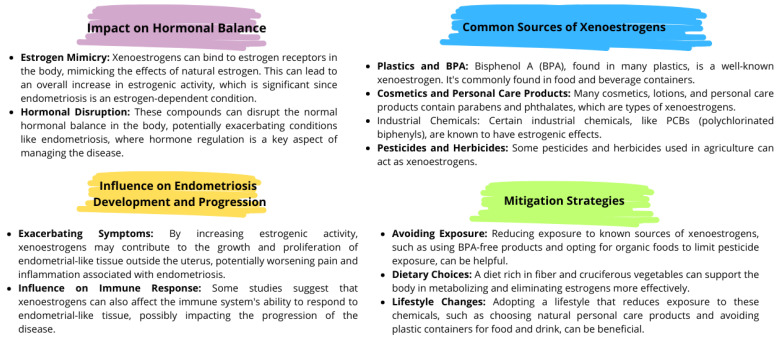
The effect of xenoestrogens on endometriosis; based on [167,168,169,170,171].

Xenoestrogens are a type of environmental estrogen consisting of synthetically produced compounds that imitate the action of the natural hormone estrogen in the body. These chemicals can bind to estrogen receptors and mimic or block the effects of natural estrogens. Their presence and activity in the human body may disrupt normal hormonal balance, leading to various health problems [167,168,169] (Figure 5). They can be found in a variety of industrial, agricultural, and consumer products such as plastics, cosmetics, personal care products, pesticides, and herbicides. Their role in the course of endometriosis is increasingly being investigated due to their potential impact on hormonal balance and reproductive health. Xenoestrogens can bind to estrogen receptors in the body, mimicking the effects of natural estrogen. This may lead to an overall increase in estrogen activity. By increasing estrogenic activity, xenoestrogens may promote the growth and proliferation of endometrial-like tissue outside the uterus, potentially worsening the pain and inflammation associated with endometriosis. Some research suggests that xenoestrogens may also affect the immune system’s ability to respond to endometrial-like tissue, possibly affecting disease progression [170,171].

Although research is ongoing and a direct causal link between xenoestrogens and endometriosis is still being established, there is increasing consensus regarding the potential impact of these environmental hormones on this disease [156]. Given the estrogen-dependent nature of endometriosis, controlling xenoestrogen exposure may be an important aspect of a comprehensive approach to the treatment and management of this disease. However, it is important to remember that avoiding xenoestrogens is only one part of a broader strategy that should also include treatment, lifestyle changes, and nutritional support.

## 4. Conclusions

The current research highlights the importance of nutrition in the treatment of endometriosis. Although many vitamins as well as micro and macro elements influence the course of the disease and its symptoms, not all connections are fully understood. Studying the impact of food on the course of endometriosis is important for several reasons, primarily because diet can significantly influence the symptoms, progression, and overall treatment of this disease. We believe that further clinical research on nutrition in endometriosis may contribute to creating recommendations for diet modification. Additionally, exploring this topic will allow patients to have the most up-to-date knowledge, which will help them make appropriate dietary modifications. Research in this area not only helps develop effective nutritional guidelines for people with endometriosis but also contributes to a broader understanding of the pathophysiology of the disease. Given the complexity and individual differences in endometriosis, a detailed understanding of the impact of diet on its course is invaluable to both healthcare professionals and patients in the treatment of this disease.

## Figures and Tables

**Table 1:** Effects of vitamin D application on endometriosis as presented in animal and in vitro studies.

Proposed Targets of Vitamin D Action	Authors and Year of the Study	Study Groups	Supplementation Dose of Vitamin D	Detailed Observed Changes	Possible Mechanisms of Vitamin D Action
Size of the endometriotic lesions	Burjiah et al., 2022 [44]	24 mice divided into four equal groups	supplementation with 8, 16, or 24 IU of vitamin D for 3 weeks	decreased size of the lesion	mechanism of action was not indicated
	Abbas et al., 2013 [45]	21 rats divided into three equal groups	supplementation with 42 μg/kg of cholecalciferol	decreased cross-sectional area of the lesions	mechanism of action was not indicated
	Yildirim et al., 2014 [46]	21 rats divided into three equal groups	supplementation with 0.05 μg/kg of 1,25(OH)_2_D_3_ for 4 weeks in 2 groups	decreased weight and decreased volume of lesions	mechanism of action was not indicated
	Akyol et al., 2016 [47]	30 rats divided into three equal groups	supplementation with 42 μg/kg per day of cholecalciferol	no observed changes	-
Matrix Metalloproteinases (MMPs)	Yildrim et al., 2014 [46]	21 rats divided into three equal groups	supplementation with 0.05 μg/kg of 1,25(OH)_2_D_3_ for 4 weeks in 2 groups	decreased MMP-9 expression	mechanism of action was not indicated
	Miyashita et al., 2016 [48]	isolated human endometriotic stromal cells (hESCs) isolated from 7 patients with endometriosis	incubation with 10^9^ or 10^7^ M 1,25(OH)_2_D_3_	decreased MMP-9 and MMP-2 expression	mechanism of action was not indicated
	Ingles et al., 2017 [49]	human endometriotic stromal cells (43 tissue samples)	incubation with 0.1 μM 1,25(OH)_2_D_3_	decreased MMP production	upregulation of the Matrix Metalloproteinase Inhibition pathway
Proinflammatory molecules	Burjiah et al., 2022 [44]	24 mice divided into four equal groups	supplementation with 8, 16, or 24 IU of vitamin D for 3 weeks	reduced inflammation	decreased production of IL-17
	Akyol et al., 2016 [47]	30 rats divided into three equal groups	supplementation with 42 μg/kg per day of cholecalciferol	reduced inflammation	decreased production of IL-6
	Delbandi et al., 2016 [50]	human endometriotic stromal cells (hESCs) isolated from 25 patients with endometriosis	10^7^ M 1,25(OH)_2_ vitamin D_3_	reduced inflammation	decreased production of IL-6
	Miyashita et al., 2016 [48]	isolated human endometriotic stromal cells (hESCs) isolated from 7 patients with endometriosis	incubation with 10^9^ or 10^7^ M 1,25(OH)_2_D_3_	reduced inflammation	decreased production of IL-8; decreased production of PGE2; decreased expression of COX-2 mRNA; decreased expression of mPGES1 mRNA; decreased expression of mPGES2 mRNA
Angiogenesis	Delbandi et al., 2016 [50]	human endometriotic stromal cells (hESCs) isolated from 25 patients with endometriosis	10^7^ M 1,25(OH)_2_ vitamin D_3_	decreased angiogenesis	decreased VEGF-A gene expression
	Miyashita et al., 2016 [48]	human endometriotic stromal cells (hESCs) isolated from 7 patients with endometriosis	incubation with 10^9^ or 10^7^ M 1,25(OH)_2_D_3_	decreased angiogenesis	inhibition of NF-κB pathway
Invasion	Delbandi et al., 2016 [50]	human endometriotic stromal cells (hESCs) isolated from 25 patients with endometriosis	10^7^ M 1,25(OH)_2_ vitamin D_3_	reduced invasion, shown as a lower ability of endometriotic cells to digest and migration through the membrane	mechanism of action was not indicated
	Pazhohan et al., 2018 [51]	blood, endometrial fluid, and tissue samples isolated from 16 patients with endometriosis	supplementation with 50,000 IU of vitamin D weekly for 12–14 weeks	decreased endometriosis cell invasion	elevated expression of CD44 glycoprotein
Proliferation	Delbandi et al., 2016 [50]	human endometriotic stromal cells (hESCs) isolated from 25 patients with endometriosis	10^7^ M 1,25(OH)_2_ vitamin D_3_	decreased proliferation of endometriosis cells	mechanism of action was not indicated
	Miyashita et al., 2016 [48]	human endometriotic stromal cells (hESCs) isolated from 7 patients with endometriosis	incubation with 10^9^ or 10^7^ M 1,25(OH)_2_D_3_	decreased proliferation of endometriosis cells	suppressed inhibition of IκBα, leading to reduction of NF-κB pathway activity
	Inges et al., 2017 [49]	human endometriotic stromal cells (43 tissue samples)	incubation with 0.1 μM 1,25(OH)_2_D_3_	decreased proliferation of endometriosis cells	down-regulation of genes involved in the axonal guidance pathway
	Pazhohan et al., 2021 [51]	blood, endometrial fluid, and tissue samples isolated from 16 patients with endometriosis	supplementation with 50,000 IU of vitamin D weekly for 12–14 weeks	decreased proliferation of endometriosis cells	reduced activity of β-catenin
Apoptosis	Abbas et al., 2013 [45]	21 rats divided into three equal groups	supplementation with 42 μg/kg of cholecalciferol	increased apoptosis of endometriosis cells	increased number of apoptotic cells
	Miyashita et al., 2016 [48]	human endometriotic stromal cells (hESCs) isolated from 7 patients with endometriosis	incubation with 10^9^ or 10^7^ M 1,25(OH)_2_D_3_	no observed changes	-
	Delbandi et al., 2016 [50]	human endometriotic stromal cells (hESCs) isolated from 25 patients with endometriosis	10^7^ M 1,25(OH)_2_ vitamin D_3_	no observed changes	-
	Rashidi et al., 2023 [52]	human endometrial stromal cells (hESCs) isolated from 10 women with endometriosis	incubation with 10 nmol/L 1,25(OH)_2_D_3_	increased apoptosis of endometriosis cells	arresting of endometriosis cells into phase G0/G1 of the cell cycle

**Table 2:** The preclinical studies evaluating the role of omega-3-PUFAs in the pathogenesis of endometriosis.

Authors and Year of the Study	Type of the Study	Applied Intervention	Main Results
Akyol et al., 2016 [47]	preclinical animal	supplementation with vitamin D and omega-3-PUFAs in rats	omega-3-PUFA supplementation reduced lesion volumes omega-3-PUFA supplementation reduced IL-6, TNF-α, and VEGF concentrations in peritoneal fluid
Tomio et al., 2013 [141]	preclinical animal	use of transgenic mice able to convert omega-6-PUFAs to omega-3-PUFAs and mice without such properties	increased omega-3-PUFA concentrations resulted in the reduction of the number and the volume of endometriosis implants and reduced the levels of IL-6
Attaman et al., 2014 [142]	preclinical animal	use of transgenic mice able to convert omega-6-PUFAs to omega-3-PUFAs and mice without such properties	increased omega-3-PUFA concentrations resulted in the reduction of COX-2 Ph-3-mitotic marker expression
Herington et al., 2013 [143]	preclinical animal	supplementation with fish oil	omega-3-PUFA supplementation reduced the adhesion formation and collagen deposition

## Data Availability

Not applicable.

## References

[1] Zondervan K.T., Becker C.M., Missmer S.A. (2020). Endometriosis. N. Engl. J. Med..

[2] Kalaitzopoulos D.R., Lempesis I.G., Samartzis N., Kolovos G., Dedes I., Daniilidis A., Nirgianakis K., Leeners B., Goulis D.G., Samartzis E.P. (2021). Leptin Concentrations in Endometriosis: A Systematic Review and Meta-Analysis. J. Reprod. Immunol..

[3] Bjørklund G., Aaseth J., Doşa M.D., Pivina L., Dadar M., Pen J.J., Chirumbolo S. (2019). Does Diet Play a Role in Reducing Nociception Related to Inflammation and Chronic Pain?. Nutrition.

[4] Alesi S., Villani A., Mantzioris E., Takele W.W., Cowan S., Moran L.J., Mousa A. (2022). Anti-Inflammatory Diets in Fertility: An Evidence Review. Nutrients.

[5] Fabozzi G., Verdone G., Allori M., Cimadomo D., Tatone C., Stuppia L., Franzago M., Ubaldi N., Vaiarelli A., Ubaldi F.M. (2022). Personalized Nutrition in the Management of Female Infertility: New Insights on Chronic Low-Grade Inflammation. Nutrients.

[6] Krabbenborg I., De Roos N., Van Der Grinten P., Nap A. (2021). Diet Quality and Perceived Effects of Dietary Changes in Dutch Endometriosis Patients: An Observational Study. Reprod. BioMed. Online.

[7] Armour M., Sinclair J., Chalmers K.J., Smith C.A. (2019). Self-Management Strategies amongst Australian Women with Endometriosis: A National Online Survey. BMC Complement. Altern. Med..

[8] Armour M., Middleton A., Lim S., Sinclair J., Varjabedian D., Smith C.A. (2021). Dietary Practices of Women with Endometriosis: A Cross-Sectional Survey. J. Altern. Complement. Med..

[9] Becker C.M., Bokor A., Heikinheimo O., Horne A., Jansen F., Kiesel L., King K., Kvaskoff M., Nap A., Petersen K. (2022). ESHRE Guideline: Endometriosis. Human. Reprod. Open.

[10] Interdonato L., Siracusa R., Fusco R., Cuzzocrea S., Di Paola R. (2023). Endocrine Disruptor Compounds in Environment: Focus on Women’s Reproductive Health and Endometriosis. Int. J. Mol. Sci..

[11] Ek M., Roth B., Ekström P., Valentin L., Bengtsson M., Ohlsson B. (2015). Gastrointestinal Symptoms among Endometriosis Patients—A Case-Cohort Study. BMC Women’s Health.

[12] Smolarz B., Szyłło K., Romanowicz H. (2021). Endometriosis: Epidemiology, Classification, Pathogenesis, Treatment and Genetics (Review of Literature). Int. J. Mol. Sci..

[13] Agostinis C., Balduit A., Mangogna A., Zito G., Romano F., Ricci G., Kishore U., Bulla R. (2021). Immunological Basis of the Endometriosis: The Complement System as a Potential Therapeutic Target. Front. Immunol..

[14] Habib N., Buzzaccarini G., Centini G., Moawad G., Ceccaldi P.-F., Gitas G., Alkatout I., Gullo G., Terzic S., Sleiman Z. (2022). Impact of Lifestyle and Diet on Endometriosis: A Fresh Look to a Busy Corner. Menopause Rev..

[15] Roshanzadeh G., Jahanian Sadatmahalleh S., Moini A., Mottaghi A., Rostami F. (2023). The Relationship between Dietary Micronutrients and Endometriosis: A Case-Control Study. Int. J. Reprod. Biomed..

[16] Yalçın Bahat P., Ayhan I., Üreyen Özdemir E., İnceboz Ü., Oral E. (2022). Dietary Supplements for Treatment of Endometriosis: A Review. Acta Biomed. Atenei Parm..

[17] Jurkiewicz-Przondziono J., Lemm M., Kwiatkowska-Pamuła A., Ziółko E., Wójtowicz M.K. (2017). Influence of Diet on the Risk of Developing Endometriosis. Ginekol. Pol..

[18] Barnard N.D., Holtz D.N., Schmidt N., Kolipaka S., Hata E., Sutton M., Znayenko-Miller T., Hazen N.D., Cobb C., Kahleova H. (2023). Nutrition in the Prevention and Treatment of Endometriosis: A Review. Front. Nutr..

[19] Monnin N., Fattet A.J., Koscinski I. (2023). Endometriosis: Update of Pathophysiology, (Epi) Genetic and Environmental Involvement. Biomedicines.

[20] Cirillo M., Argento F.R., Becatti M., Fiorillo C., Coccia M.E., Fatini C. (2023). Mediterranean Diet and Oxidative Stress: A Relationship with Pain Perception in Endometriosis. Int. J. Mol. Sci..

[21] Hondal R.J. (2023). Selenium Vitaminology: The Connection between Selenium, Vitamin C, Vitamin E, and Ergothioneine. Curr. Opin. Chem. Biol..

[22] Carr A., Maggini S. (2017). Vitamin C and Immune Function. Nutrients.

[23] Padayatty S., Levine M. (2016). Vitamin C: The Known and the Unknown and Goldilocks. Oral. Dis..

[24] Spoelstra-de Man A.M.E., Elbers P.W.G., Oudemans-Van Straaten H.M. (2018). Vitamin C: Should We Supplement?. Curr. Opin. Crit. Care.

[25] Lee G., Han S. (2018). The Role of Vitamin E in Immunity. Nutrients.

[26] Miyazawa T., Burdeos G.C., Itaya M., Nakagawa K., Miyazawa T. (2019). Vitamin E: Regulatory Redox Interactions: VITAMIN E: REGULATORY REDOX INTERACTIONS. IUBMB Life.

[27] Da Broi M.G., De Albuquerque F.O., De Andrade A.Z., Cardoso R.L., Jordão Junior A.A., Navarro P.A. (2016). Increased Concentration of 8-Hydroxy-2′-Deoxyguanosine in Follicular Fluid of Infertile Women with Endometriosis. Cell Tissue Res..

[28] Da Broi M.G., Jordão-Jr A.A., Ferriani R.A., Navarro P.A. (2018). Oocyte Oxidative DNA Damage May Be Involved in Minimal/Mild Endometriosis-Related Infertility. Mol. Reprod. Dev..

[29] Ferreira E.M., Giorgi V.S.I., Rodrigues J.K., De Andrade A.Z., Junior A.A.J., Navarro P.A. (2019). Systemic Oxidative Stress as a Possible Mechanism Underlying the Pathogenesis of Mild Endometriosis-Related Infertility. Reprod. BioMed. Online.

[30] Liu F., He L., Liu Y., Shi Y., Du H. (2013). The Expression and Role of Oxidative Stress Markers in the Serum and Follicular Fluid of Patients with Endometriosis. Clin. Exp. Obstet. Gynecol..

[31] Ekici E.İ., Güney M., Nazıroğlu M. (2020). Protective Effect of Cabergoline on Mitochondrial Oxidative Stress-Induced Apoptosis Is Mediated by Modulations of TRPM2 in Neutrophils of Patients with Endometriosis. J. Bioenerg. Biomembr..

[32] Lu X., Wu Z., Wang M., Cheng W. (2018). Effects of Vitamin C on the Outcome of in Vitro Fertilization–Embryo Transfer in Endometriosis: A Randomized Controlled Study. J. Int. Med. Res..

[33] Nishihara T., Matsumoto K., Hosoi Y., Morimoto Y. (2018). Evaluation of Antioxidant Status and Oxidative Stress Markers in Follicular Fluid for Human in Vitro Fertilization Outcome. Reprod. Med. Biol..

[34] Hoorsan H., Simbar M., Tehrani F.R., Fathi F., Mosaffa N., Riazi H., Akradi L., Nasseri S., Bazrafkan S. (2022). The Effectiveness of Antioxidant Therapy (Vitamin C) in an Experimentally Induced Mouse Model of Ovarian Endometriosis. Womens Health.

[35] Erten O.U., Ensari T.A., Dilbaz B., Cakiroglu H., Altinbas S.K., Çaydere M., Goktolga U. (2016). Vitamin C Is Effective for the Prevention and Regression of Endometriotic Implants in an Experimentally Induced Rat Model of Endometriosis. Taiwan. J. Obstet. Gynecol..

[36] Durak Y., Kokcu A., Kefeli M., Bildircin D., Çelik H., Alper T. (2013). Effect of Vitamin C on the Growth of Experimentally Induced Endometriotic Cysts: Vitamin C and Endometriosis Development. J. Obstet. Gynaecol. Res..

[37] Dai Y., Lin X., Xu W., Lin X., Huang Q., Shi L., Pan Y., Zhang Y., Zhu Y., Li C. (2019). MiR-210-3p Protects Endometriotic Cells from Oxidative Stress-Induced Cell Cycle Arrest by Targeting BARD1. Cell Death Dis..

[38] Amini L., Chekini R., Nateghi M.R., Haghani H., Jamialahmadi T., Sathyapalan T., Sahebkar A. (2021). The Effect of Combined Vitamin C and Vitamin E Supplementation on Oxidative Stress Markers in Women with Endometriosis: A Randomized, Triple-Blind Placebo-Controlled Clinical Trial. Pain. Res. Manag..

[39] Santanam N., Kavtaradze N., Murphy A., Dominguez C., Parthasarathy S. (2013). Antioxidant Supplementation Reduces Endometriosis-Related Pelvic Pain in Humans. Transl. Res..

[40] Darling A.M., Chavarro J.E., Malspeis S., Harris H.R., Missmer S.A. (2013). A Prospective Cohort Study of Vitamins B, C, E, and Multivitamin Intake and Endometriosis. J. Endometr. Pelvic Pain. Disord..

[41] Schink M., Konturek P.C., Herbert S.L., Renner S.P., Burghaus S., Blum S., Fasching P.A., Neurath M.F., Zopf Y. (2019). Different Nutrient Intake and Prevalence of Gastrointestinal Comorbidities in Women with Endometriosis. J. Physiol. Pharmacol..

[42] Delrue C., Speeckaert M.M. (2023). Vitamin D and Vitamin D-Binding Protein in Health and Disease. Int. J. Mol. Sci..

[43] Ghanavatinejad A., Rashidi N., Mirahmadian M., Rezania S., Mosalaei M., Ghasemi J., Zarnani A.-H. (2021). Vitamin D3 Controls TLR4- and TLR2-Mediated Inflammatory Responses of Endometrial Cells. Gynecol. Obstet. Investig..

[44] Burjiah A., Adi A., Widjiati W. (2022). Vitamin D Inhibited Endometriosis Development in Mice Model through Interleukin 17 Modulation. Open Vet. J..

[45] Abbas M.A., Taha M.O., Disi A.M., Shomaf M. (2013). Regression of Endometrial Implants Treated with Vitamin D3 in a Rat Model of Endometriosis. Eur. J. Pharmacol..

[46] Yildirim B., Guler T., Akbulut M., Oztekin O., Sariiz G. (2014). 1–Alpha, 25–Dihydroxyvitamin D3 Regresses Endometriotic Implants in Rats by Inhibiting Neovascularization and Altering Regulation of Matrix Metalloproteinase. Postgrad. Med..

[47] Akyol A., Şimşek M., İlhan R., Can B., Baspinar M., Akyol H., Gül H.F., Gürsu F., Kavak B., Akın M. (2016). Efficacies of Vitamin D and Omega-3 Polyunsaturated Fatty Acids on Experimental Endometriosis. Taiwan J. Obstet. Gynecol..

[48] Miyashita M., Koga K., Izumi G., Sue F., Makabe T., Taguchi A., Nagai M., Urata Y., Takamura M., Harada M. (2016). Effects of 1,25-Dihydroxy Vitamin D3 on Endometriosis. J. Clin. Endocrinol. Metab..

[49] Ingles S.A., Wu L., Liu B.T., Chen Y., Wang C.-Y., Templeman C., Brueggmann D. (2017). Differential Gene Expression by 1,25(OH) 2D3 in an Endometriosis Stromal Cell Line. J. Steroid Biochem. Mol. Biol..

[50] Delbandi A.-A., Mahmoudi M., Shervin A., Zarnani A.-H. (2016). 1,25-Dihydroxy Vitamin D3 Modulates Endometriosis-Related Features of Human Endometriotic Stromal Cells. Am. J. Reprod. Immunol..

[51] Pazhohan A., Amidi F., Akbari-Asbagh F., Seyedrezazadeh E., Aftabi Y., Abdolalizadeh J., Khodarahmian M., Khanlarkhani N., Sobhani A. (2018). Expression and Shedding of CD44 in the Endometrium of Women with Endometriosis and Modulating Effects of Vitamin D: A Randomized Exploratory Trial. J. Steroid Biochem. Mol. Biol..

[52] Rashidi N., Arefi S., Sadri M., Delbandi A.-A. (2023). Effect of Active Vitamin D on Proliferation, Cell Cycle and Apoptosis in Endometriotic Stromal Cells. Reprod. BioMed. Online.

[53] Ke J., Ye J., Li M., Zhu Z. (2021). The Role of Matrix Metalloproteinases in Endometriosis: A Potential Target. Biomolecules.

[54] Pazhohan A., Danaei-Mehrabad S., Mohamad-Rezaeii Z., Amidi F., Khodarahmian M., Shabani Nashtaei M., Sobhani A., Farajzadeh M.A. (2021). The Modulating Effects of Vitamin D on the Activity of β-Catenin in the Endometrium of Women with Endometriosis: A Randomized Exploratory Trial. Gynecol. Endocrinol..

[55] Ma B., Hottiger M.O. (2016). Crosstalk between Wnt/β-Catenin and NF-κB Signaling Pathway during Inflammation. Front. Immunol..

[56] Yarmolinskaya M., Denisova A., Tkachenko N., Ivashenko T., Bespalova O., Tolibova G., Tral T. (2021). Vitamin D Significance in Pathogenesis of Endometriosis. Gynecol. Endocrinol..

[57] Delbandi A.-A., Torab M., Abdollahi E., Khodaverdi S., Rokhgireh S., Moradi Z., Heidari S., Mohammadi T. (2021). Vitamin D Deficiency as a Risk Factor for Endometriosis in Iranian Women. J. Reprod. Immunol..

[58] Anastasi E., Fuggetta E., De Vito C., Migliara G., Viggiani V., Manganaro L., Granato T., Benedetti Panici P., Angeloni A., Porpora M.G. (2017). Low Levels of 25-OH Vitamin D in Women with Endometriosis and Associated Pelvic Pain. Clin. Chem. Lab. Med. (CCLM).

[59] Buggio L., Somigliana E., Pizzi M.N., Dridi D., Roncella E., Vercellini P. (2019). 25-Hydroxyvitamin D Serum Levels and Endometriosis: Results of a Case–Control Study. Reprod. Sci..

[60] Dressler N., Chandra A., Aguirre Dávila L., Spineli L.M., Schippert C., Von Versen-Höynck F. (2016). BMI and Season Are Associated with Vitamin D Deficiency in Women with Impaired Fertility: A Two-Centre Analysis. Arch. Gynecol. Obstet..

[61] Ciavattini A., Serri M., Delli Carpini G., Morini S., Clemente N. (2017). Ovarian Endometriosis and Vitamin D Serum Levels. Gynecol. Endocrinol..

[62] Mehdizadehkashi A., Rokhgireh S., Tahermanesh K., Eslahi N., Minaeian S., Samimi M. (2021). The Effect of Vitamin D Supplementation on Clinical Symptoms and Metabolic Profiles in Patients with Endometriosis. Gynecol. Endocrinol..

[63] Nodler J.L., DiVasta A.D., Vitonis A.F., Karevicius S., Malsch M., Sarda V., Fadayomi A., Harris H.R., Missmer S.A. (2020). Supplementation with Vitamin D or ω-3 Fatty Acids in Adolescent Girls and Young Women with Endometriosis (SAGE): A Double-Blind, Randomized, Placebo-Controlled Trial. Am. J. Clin. Nutr..

[64] Almassinokiani F., Khodaverdi S., Solaymani-dodaran M., Akbari P., Pazouki A. (2016). Effects of Vitamin D on Endometriosis-Related Pain: A Double-Blind Clinical Trial. Med. Sci. Monit..

[65] Shim J.Y., Laufer M.R., King C.R., Lee T.T.M., Einarsson J.I., Tyson N. (2024). Evaluation and Management of Endometriosis in the Adolescent. Obstet. Gynecol..

[66] Pierzchalski K., Taylor R.N., Nezhat C., Jones J.W., Napoli J.L., Yang G., Kane M.A., Sidell N. (2014). Retinoic Acid Biosynthesis Is Impaired in Human and Murine Endometriosis1. Biol. Reprod..

[67] Yamagata Y., Takaki E., Shinagawa M., Okada M., Jozaki K., Lee L., Sato S., Maekawa R., Taketani T., Asada H. (2015). Retinoic Acid Has the Potential to Suppress Endometriosis Development. J. Ovarian Res..

[68] Samavat H., Kurzer M.S. (2015). Estrogen Metabolism and Breast Cancer. Cancer Lett..

[69] Li S., Fu X., Wu T., Yang L., Hu C., Wu R. (2017). Role of Interleukin-6 and Its Receptor in Endometriosis. Med. Sci. Monit..

[70] Li L., Gao H., Pan L., Zhao Y., Liang Z., Zhang Q., Wang D. (2021). All-Trans Retinoic Acid Inhibits Epithelial-to-Mesenchymal Transition (EMT) through the down-Regulation of IL-6 in Endometriosis. Ann. Palliat. Med..

[71] Czuba L.C., Zhong G., Yabut K.C., Isoherranen N. (2020). Analysis of Vitamin A and Retinoids in Biological Matrices. Methods in Enzymology.

[72] Hanna M., Jaqua E., Nguyen V., Clay J. (2022). B Vitamins: Functions and Uses in Medicine. Perm. J..

[73] Gille D., Schmid A. (2015). Vitamin B12 in Meat and Dairy Products. Nutr. Rev..

[74] Ali M.A., Hafez H.A., Kamel M.A., Ghamry H.I., Shukry M., Farag M.A. (2022). Dietary Vitamin B Complex: Orchestration in Human Nutrition throughout Life with Sex Differences. Nutrients.

[75] Pattanittum P., Kunyanone N., Brown J., Sangkomkamhang U.S., Barnes J., Seyfoddin V., Marjoribanks J. (2016). Dietary Supplements for Dysmenorrhoea. Cochrane Database Syst. Rev..

[76] Abdollahifard S., Rahmanian Koshkaki A., Moazamiyanfar R. (2014). The Effects of Vitamin B1 on Ameliorating the Premenstrual Syndrome Symptoms. Glob. J. Health Sci..

[77] Farag M.A., Abib B., Qin Z., Ze X., Ali S.E. (2023). Dietary Macrominerals: Updated Review of Their Role and Orchestration in Human Nutrition throughout the Life Cycle with Sex Differences. Curr. Res. Food Sci..

[78] Ali A.A.H. (2023). Overview of the Vital Roles of Macro Minerals in the Human Body. J. Trace Elem. Miner..

[79] Weyh C., Krüger K., Peeling P., Castell L. (2022). The Role of Minerals in the Optimal Functioning of the Immune System. Nutrients.

[80] Markowska A., Antoszczak M., Markowska J., Huczyński A. (2023). The Role of Selected Dietary Factors in the Development and Course of Endometriosis. Nutrients.

[81] Dubey P., Thakur V., Chattopadhyay M. (2020). Role of Minerals and Trace Elements in Diabetes and Insulin Resistance. Nutrients.

[82] Delsouc M.B., Ghersa F., Ramírez D., Della Vedova M.C., Gil R.A., Vallcaneras S.S., Casais M. (2019). Endometriosis Progression in Tumor Necrosis Factor Receptor P55-Deficient Mice: Impact on Oxidative/Nitrosative Stress and Metallomic Profile. J. Trace Elem. Med. Biol..

[83] Dring J.C., Forma A., Chilimoniuk Z., Dobosz M., Teresiński G., Buszewicz G., Flieger J., Cywka T., Januszewski J., Baj J. (2021). Essentiality of Trace Elements in Pregnancy, Fertility, and Gynecologic Cancers—A State-of-the-Art Review. Nutrients.

[84] Maret W. (2013). Zinc Biochemistry: From a Single Zinc Enzyme to a Key Element of Life. Adv. Nutr..

[85] Laganà A.S., Garzon S., Götte M., Viganò P., Franchi M., Ghezzi F., Martin D.C. (2019). The Pathogenesis of Endometriosis: Molecular and Cell Biology Insights. Int. J. Mol. Sci..

[86] Yang Y.-M., Yang W.-X. (2017). Epithelial-to-Mesenchymal Transition in the Development of Endometriosis. Oncotarget.

[87] Debnath P., Huirem R.S., Dutta P., Palchaudhuri S. (2022). Epithelial–Mesenchymal Transition and Its Transcription Factors. Biosci. Rep..

[88] Lamouille S., Xu J., Derynck R. (2014). Molecular Mechanisms of Epithelial–Mesenchymal Transition. Nat. Rev. Mol. Cell Biol..

[89] Kapoor C., Vaidya S., Wadhwan V., Hitesh, Kaur G., Pathak A. (2016). Seesaw of Matrix Metalloproteinases (MMPs). J. Can. Res. Ther..

[90] Huang L., Drake V.J., Ho E. (2015). Zinc. Adv. Nutr..

[91] Singh A.K., Chattopadhyay R., Chakravarty B., Chaudhury K. (2013). Markers of Oxidative Stress in Follicular Fluid of Women with Endometriosis and Tubal Infertility Undergoing IVF. Reprod. Toxicol..

[92] Di Carlo C., Bonifacio M., Tommaselli G.A., Bifulco G., Guerra G., Nappi C. (2009). Metalloproteinases, Vascular Endothelial Growth Factor, and Angiopoietin 1 and 2 in Eutopic and Ectopic Endometrium. Fertil. Steril..

[93] Malvezzi H., Aguiar V.G., de Paz C.C.P., Tanus-Santos J.E., Penna I.A.d.A., Navarro P.A. (2013). Increased Circulating MMP-2 Levels in Infertile Patients With Moderate and Severe Pelvic Endometriosis. Reprod. Sci..

[94] Cao J., Duan S., Zhang H., Chen Y., Guo M. (2020). Zinc Deficiency Promoted Fibrosis via ROS and TIMP/MMPs in the Myocardium of Mice. Biol. Trace Elem. Res..

[95] Scrimgeour A.G., Carrigan C.T., Condlin M.L., Urso M.L., Van Den Berg R.M., Van Helden H.P.M., Montain S.J., Joosen M.J.A. (2018). Dietary Zinc Modulates Matrix Metalloproteinases in Traumatic Brain Injury. J. Neurotrauma.

[96] Furuya M., Masuda H., Hara K., Uchida H., Sato K., Sato S., Asada H., Maruyama T., Yoshimura Y., Katabuchi H. (2017). ZEB1 Expression Is a Potential Indicator of Invasive Endometriosis. Acta Obstet. Gynecol. Scand..

[97] Bartnik P., Kacperczyk-Bartnik J., Goławski K., Sierdziński J., Mańka G., Kiecka M., Lipa M., Warzecha D., Spaczyński R., Piekarski P. (2022). Plasma and Peritoneal Fluid ZEB Levels in Patients with Endometriosis and Infertility. Biomedicines.

[98] Messalli E.M., Schettino M.T., Mainini G., Ercolano S., Fuschillo G., Falcone F., Esposito E., Di Donna M.C., De Franciscis P., Torella M. (2014). The Possible Role of Zinc in the Etiopathogenesis of Endometriosis. Clin. Exp. Obstet. Gynecol..

[99] Lai G.-L., Yeh C.-C., Yeh C.-Y., Chen R.-Y., Fu C.-L., Chen C.-H., Tzeng C.-R. (2017). Decreased Zinc and Increased Lead Blood Levels Are Associated with Endometriosis in Asian Women. Reprod. Toxicol..

[100] Jeon Y., Yoon J.D., Cai L., Hwang S.-U., Kim E., Zheng Z., Lee E., Kim D.Y., Hyun S.-H. (2014). Supplementation of Zinc on Oocyte in Vitro Maturation Improves Preimplatation Embryonic Development in Pigs. Theriogenology.

[101] Duncan F.E., Que E.L., Zhang N., Feinberg E.C., O’Halloran T.V., Woodruff T.K. (2016). The Zinc Spark Is an Inorganic Signature of Human Egg Activation. Sci. Rep..

[102] Bost M., Houdart S., Oberli M., Kalonji E., Huneau J.-F., Margaritis I. (2016). Dietary Copper and Human Health: Current Evidence and Unresolved Issues. J. Trace Elem. Med. Biol..

[103] Kaleler İ., Acikgoz A.S., Gezer A., Uslu E. (2021). A Potential Role of Sirtuin3 and Its Target Enzyme Activities in Patients with Ovarian Endometrioma. Gynecol. Endocrinol..

[104] Byrne C., Divekar S.D., Storchan G.B., Parodi D.A., Martin M.B. (2013). Metals and Breast Cancer. J. Mammary Gland. Biol. Neoplasia.

[105] Thézénas M.-L., De Leo B., Laux-Biehlmann A., Bafligil C., Elger B., Tapmeier T., Morten K., Rahmioglu N., Dakin S.G., Charles P. (2020). Amine Oxidase 3 Is a Novel Pro-Inflammatory Marker of Oxidative Stress in Peritoneal Endometriosis Lesions. Sci. Rep..

[106] Turgut A., Özler A., Görük N.Y., Tunc S.Y., Evliyaoglu O., Gül T. (2013). Copper, Ceruloplasmin and Oxidative Stress in Patients with Advanced-Stage Endometriosis. Eur. Rev. Med. Pharmacol. Sci..

[107] Ergaz Z., Weinstein-Fudim L., Ornoy A. (2018). High Sucrose Low Copper Diet in Pregnant Diabetic Rats Induces Transient Oxidative Stress, Hypoxia, and Apoptosis in the Offspring’s Liver. Birth Defects Res..

[108] Pollack A.Z., Louis G.M.B., Chen Z., Peterson C.M., Sundaram R., Croughan M.S., Sun L., Hediger M.L., Stanford J.B., Varner M.W. (2013). Trace Elements and Endometriosis: The ENDO Study. Reprod. Toxicol..

[109] Singh A., Ghosh P., Mukherjee S., Ojha A.K., Hansda A., Choudhury P., Halder S., Sharma S., Mukherjee G., Dasgupta S. (2022). Transition Metallo-Curcumin Complexes: A New Hope for Endometriosis?. J. Mater. Chem. B.

[110] Ni S., Yuan Y., Kuang Y., Li X. (2022). Iron Metabolism and Immune Regulation. Front. Immunol..

[111] Ansariniya H., Yavari A., Javaheri A., Zare F. (2022). Oxidative Stress-related Effects on Various Aspects of Endometriosis. Am. J. Rep. Immunol..

[112] Scutiero G., Iannone P., Bernardi G., Bonaccorsi G., Spadaro S., Volta C.A., Greco P., Nappi L. (2017). Oxidative Stress and Endometriosis: A Systematic Review of the Literature. Oxidative Med. Cell. Longev..

[113] Pascolo L., Pachetti M., Camillo A., Cernogoraz A., Rizzardi C., Mikus K.V., Zanconati F., Salomé M., Suárez V.T., Romano F. (2023). Detention and Mapping of Iron and Toxic Environmental Elements in Human Ovarian Endometriosis: A Suggested Combined Role. Sci. Total Environ..

[114] Imanaka S., Yamada Y., Kawahara N., Kobayashi H. (2021). A Delicate Redox Balance between Iron and Heme Oxygenase-1 as an Essential Biological Feature of Endometriosis. Arch. Med. Res..

[115] Polak G., Barczyński B., Wertel I., Kwaśniewski W., Bednarek W., Derewianka-Polak M., Frąszczak K., Olajossy M., Kotarski J. (2018). Disrupted Iron Metabolism in Peritoneal Fluid May Induce Oxidative Stress in the Peritoneal Cavity of Women with Endometriosis. Ann. Agric. Environ. Med..

[116] Wu Y., Yang R., Lan J., Wu Y., Huang J., Fan Q., You Y., Lin H., Jiao X., Chen H. (2023). Iron Overload Modulates Follicular Microenvironment via ROS/HIF-1α/FSHR Signaling. Free Radic. Biol. Med..

[117] Sanchez A.M., Papaleo E., Corti L., Santambrogio P., Levi S., Vigano P., Candiani M., Panina-Bordignon P. (2014). Iron Availability Is Increased in Individual Human Ovarian Follicles in Close Proximity to an Endometrioma Compared with Distal Ones. Human. Reprod..

[118] Alizadeh M., Mahjoub S., Esmaelzadeh S., Hajian K., Basirat Z., Ghasemi M. (2015). Evaluation of Oxidative Stress in Endometriosis: A Case-Control Study. Casp. J. Intern. Med..

[119] Atiya H.I., Frisbie L., Goldfeld E., Orellana T., Donnellan N., Modugno F., Calderon M., Watkins S., Zhang R., Elishaev E. (2022). Endometriosis-Associated Mesenchymal Stem Cells Support Ovarian Clear Cell Carcinoma through Iron Regulation. Cancer Res..

[120] Imanaka S., Maruyama S., Kimura M., Nagayasu M., Kawahara N., Kobayashi H. (2021). Relationship between Cyst Fluid Concentrations of Iron and Severity of Dysmenorrhea in Patients with Ovarian Endometrioma. Gynecol. Obstet. Investig..

[121] Zhang Y., Liu X., Deng M., Xu C., Zhang Y., Wu D., Tang F., Yang R., Miao J. (2022). Ferroptosis Induced by Iron Overload Promotes Fibrosis in Ovarian Endometriosis and Is Related to Subpopulations of Endometrial Stromal Cells. Front. Pharmacol..

[122] Kieliszek M. (2019). Selenium–Fascinating Microelement, Properties and Sources in Food. Molecules.

[123] Hu W., Zhao C., Hu H., Yin S. (2021). Food Sources of Selenium and Its Relationship with Chronic Diseases. Nutrients.

[124] Avery J., Hoffmann P. (2018). Selenium, Selenoproteins, and Immunity. Nutrients.

[125] Hariharan S., Dharmaraj S. (2020). Selenium and Selenoproteins: It’s Role in Regulation of Inflammation. Inflammopharmacol.

[126] Candas D., Li J.J. (2014). MnSOD in Oxidative Stress Response-Potential Regulation via Mitochondrial Protein Influx. Antioxid. Redox Signal..

[127] Winarto H., Tan M., Sadikin M., Wanandi S. (2017). Expression Is Down-Regulated by Oxidative Stress in Endometriosis and Endometriosis-Associated Ovarian Cancer. Transl. Oncogenom..

[128] De Graaf N.P.J., Roffel S., Gibbs S., Kleverlaan C.J., Lopez Gonzalez M., Rustemeyer T., Feilzer A.J., Bontkes H.J. (2023). Nickel Allergy Is Associated with a Broad Spectrum Cytokine Response. Contact Dermat..

[129] Di Gioacchino M., Ricciardi L., De Pità O., Minelli M., Patella V., Voltolini S., Di Rienzo V., Braga M., Ballone E., Mangifesta R. (2014). Nickel Oral Hyposensitization in Patients with Systemic Nickel Allergy Syndrome. Ann. Med..

[130] Ricciardi L., Arena A., Arena E., Zambito M., Ingrassia A., Valenti G., Loschiavo G., D’Angelo A., Saitta S. (2014). Systemic Nickel Allergy Syndrome: Epidemiological Data from Four Italian Allergy Units. Int. J. Immunopathol. Pharmacol..

[131] Yuk J.-S., Kim Y.J., Yi K.-W., Tak K., Hur J.-Y., Shin J.-H. (2015). High Rate of Nickel Allergy in Women with Endometriosis: A 3-Year Population-Based Study: Nickel Allergy in Endometriosis. J. Obstet. Gynaecol. Res..

[132] Yuk J.-S., Shin J.S., Shin J.-Y., Oh E., Kim H., Park W.I. (2015). Nickel Allergy Is a Risk Factor for Endometriosis: An 11-Year Population-Based Nested Case-Control Study. PLoS ONE.

[133] Silva N., Senanayake H., Waduge V. (2013). Elevated Levels of Whole Blood Nickel in a Group of Sri Lankan Women with Endometriosis: A Case Control Study. BMC Res. Notes.

[134] Borghini R., Porpora M.G., Casale R., Marino M., Palmieri E., Greco N., Donato G., Picarelli A. (2020). Irritable Bowel Syndrome-Like Disorders in Endometriosis: Prevalence of Nickel Sensitivity and Effects of a Low-Nickel Diet. An Open-Label Pilot Study. Nutrients.

[135] Vincent J.B. (2017). New Evidence against Chromium as an Essential Trace Element. J. Nutr..

[136] Yin R.V., Phung O.J. (2015). Effect of Chromium Supplementation on Glycated Hemoglobin and Fasting Plasma Glucose in Patients with Diabetes Mellitus. Nutr. J..

[137] Vincent J.B., Lukaski H.C. (2018). Chromium. Adv. Nutr..

[138] Field C.J., Robinson L. (2019). Dietary Fats. Adv. Nutr..

[139] Djuricic I., Calder P.C. (2021). Beneficial Outcomes of Omega-6 and Omega-3 Polyunsaturated Fatty Acids on Human Health: An Update for 2021. Nutrients.

[140] Ishihara T., Yoshida M., Arita M. (2019). Omega-3 Fatty Acid-Derived Mediators That Control Inflammation and Tissue Homeostasis. Int. Immunol..

[141] Tomio K., Kawana K., Taguchi A., Isobe Y., Iwamoto R., Yamashita A., Kojima S., Mori M., Nagamatsu T., Arimoto T. (2013). Omega-3 Polyunsaturated Fatty Acids Suppress the Cystic Lesion Formation of Peritoneal Endometriosis in Transgenic Mouse Models. PLoS ONE.

[142] Attaman J.A., Stanic A.K., Kim M., Lynch M.P., Rueda B.R., Styer A.K. (2014). The Anti-Inflammatory Impact of Omega-3 Polyunsaturated Fatty Acids During the Establishment of Endometriosis-Like Lesions. Am. J. Reprod. Immunol..

[143] Herington J.L., Glore D.R., Lucas J.A., Osteen K.G., Bruner-Tran K.L. (2013). Dietary Fish Oil Supplementation Inhibits Formation of Endometriosis-Associated Adhesions in a Chimeric Mouse Model. Fertil. Steril..

[144] Stepanow K., Ogłuszka M., Lepczyński A., Poławska E., Pierzchała M. (2018). Transgeniczne Myszy Jako Model w Badaniach Wpływu Wielonienasyconych Kwasów Tłuszczowych Na Organizm. Postep. Biochem..

[145] Abd El-Kader A.I., Gonied A.S., Lotfy Mohamed M., Mohamed S.L. (2019). Impact of Endometriosis-Related Adhesions on Quality of Life among Infertile Women. Int. J. Fertil Steril..

[146] Hopeman M.M., Riley J.K., Frolova A.I., Jiang H., Jungheim E.S. (2015). Serum Polyunsaturated Fatty Acids and Endometriosis. Reprod. Sci..

[147] Kim T.-H., Jo S., Park Y., Lee H.-H., Chung S.-H., Lee W.-S. (2013). Differences in Omega-3 and Fatty Acid Profiles between Patients with Endometriosis and Those with a Functional Ovarian Cyst. J. Obstet. Gynaecol..

[148] Youseflu S., Sadatmahalleh S.J., Mottaghi A., Kazemnejad A. (2019). The Association of Food Consumption and Nutrient Intake with Endometriosis Risk in Iranian Women: A Case-Control Study. Int. J. Reprod. Biomed..

[149] Schwartz N.R.M., Afeiche M.C., Terry K.L., Farland L.V., Chavarro J.E., Missmer S.A., Harris H.R. (2022). Glycemic Index, Glycemic Load, Fiber, and Gluten Intake and Risk of Laparoscopically Confirmed Endometriosis in Premenopausal Women. J. Nutr..

[150] Markova M., Koelman L., Hornemann S., Pivovarova O., Sucher S., Machann J., Rudovich N., Thomann R., Schneeweiss R., Rohn S. (2020). Effects of Plant and Animal High Protein Diets on Immune-Inflammatory Biomarkers: A 6-Week Intervention Trial. Clin. Nutr..

[151] Nieman K.M., Anderson B.D., Cifelli C.J. (2021). The Effects of Dairy Product and Dairy Protein Intake on Inflammation: A Systematic Review of the Literature. J. Am. Coll. Nutr..

[152] Ulven S.M., Holven K.B., Gil A., Rangel-Huerta O.D. (2019). Milk and Dairy Product Consumption and Inflammatory Biomarkers: An Updated Systematic Review of Randomized Clinical Trials. Adv. Nutr..

[153] Qi X., Zhang W., Ge M., Sun Q., Peng L., Cheng W., Li X. (2021). Relationship Between Dairy Products Intake and Risk of Endometriosis: A Systematic Review and Dose-Response Meta-Analysis. Front. Nutr..

[154] Steiner B.M., Berry D.C. (2022). The Regulation of Adipose Tissue Health by Estrogens. Front. Endocrinol..

[155] Liang Z., Mahmoud Abdelshafy A., Luo Z., Belwal T., Lin X., Xu Y., Wang L., Yang M., Qi M., Dong Y. (2022). Occurrence, Detection, and Dissipation of Pesticide Residue in Plant-Derived Foodstuff: A State-of-the-Art Review. Food Chem..

[156] Wieczorek K., Szczęsna D., Jurewicz J. (2022). Environmental Exposure to Non-Persistent Endocrine Disrupting Chemicals and Endometriosis: A Systematic Review. Int. J. Environ. Res. Public Health.

[157] Youseflu S., Jahanian Sadatmahalleh S., Roshanzadeh G., Mottaghi A., Kazemnejad A., Moini A. (2020). Effects of Endometriosis on Sleep Quality of Women: Does Life Style Factor Make a Difference?. BMC Women’s Health.

[158] Yamamoto A., Harris H.R., Vitonis A.F., Chavarro J.E., Missmer S.A. (2018). A Prospective Cohort Study of Meat and Fish Consumption and Endometriosis Risk. Am. J. Obstet. Gynecol..

[159] Canivenc-Lavier M.-C., Bennetau-Pelissero C. (2023). Phytoestrogens and Health Effects. Nutrients.

[160] Bartiromo L., Schimberni M., Villanacci R., Ottolina J., Dolci C., Salmeri N., Viganò P., Candiani M. (2021). Endometriosis and Phytoestrogens: Friends or Foes? A Systematic Review. Nutrients.

[161] Szukiewicz D. (2023). Insight into the Potential Mechanisms of Endocrine Disruption by Dietary Phytoestrogens in the Context of the Etiopathogenesis of Endometriosis. Int. J. Mol. Sci..

[162] Desmawati D., Sulastri D. (2019). Phytoestrogens and Their Health Effect. Open Access Maced. J. Med. Sci..

[163] Patra S., Gorai S., Pal S., Ghosh K., Pradhan S., Chakrabarti S. (2023). A Review on Phytoestrogens: Current Status and Future Direction. Phytother. Res..

[164] Petrine J.C.P., Del Bianco-Borges B. (2021). The Influence of Phytoestrogens on Different Physiological and Pathological Processes: An Overview. Phytother. Res..

[165] Lecomte S., Demay F., Ferrière F., Pakdel F. (2017). Phytochemicals Targeting Estrogen Receptors: Beneficial Rather Than Adverse Effects?. Int. J. Mol. Sci..

[166] Cai X., Liu M., Zhang B., Zhao S.-J., Jiang S.-W. (2021). Phytoestrogens for the Management of Endometriosis: Findings and Issues. Pharmaceuticals.

[167] Wang X., Ha D., Yoshitake R., Chan Y.S., Sadava D., Chen S. (2021). Exploring the Biological Activity and Mechanism of Xenoestrogens and Phytoestrogens in Cancers: Emerging Methods and Concepts. Int. J. Mol. Sci..

[168] Paterni I., Granchi C., Minutolo F. (2017). Risks and Benefits Related to Alimentary Exposure to Xenoestrogens. Crit. Rev. Food Sci. Nutr..

[169] Wang L.-H., Chen L.-R., Chen K.-H. (2021). In Vitro and Vivo Identification, Metabolism and Action of Xenoestrogens: An Overview. Int. J. Mol. Sci..

[170] Reddy V., McCarthy M., Raval A.P. (2022). Xenoestrogens Impact Brain Estrogen Receptor Signaling during the Female Lifespan: A Precursor to Neurological Disease?. Neurobiol. Dis..

[171] Bustamante-Barrientos F.A., Méndez-Ruette M., Ortloff A., Luz-Crawford P., Rivera F.J., Figueroa C.D., Molina L., Bátiz L.F. (2021). The Impact of Estrogen and Estrogen-Like Molecules in Neurogenesis and Neurodegeneration: Beneficial or Harmful?. Front. Cell. Neurosci..

